# Mixed *Candida albicans*–*Staphylococcus aureus* Biofilm Is Reduced by Light-Activated Nanocomposite with Phloxine B

**DOI:** 10.3390/jof11080582

**Published:** 2025-08-05

**Authors:** Jarmila Czucz Varga, Juraj Bujdák, Helena Bujdáková

**Affiliations:** 1 Department of Microbiology and Virology, Faculty of Natural Sciences, Comenius University in Bratislava, Mlynská dolina, Ilkovičova 6, 842 15 Bratislava, Slovakia; vargova301@uniba.sk; 2 Department of Physical and Theoretical Chemistry, Faculty of Natural Sciences, Comenius University in Bratislava, Mlynská dolina, Ilkovičova 6, 842 15 Bratislava, Slovakia; juraj.bujdak@uniba.sk; 3 Institute of Inorganic Chemistry, Slovak Academy of Sciences, Dúbravská cesta 9, 845 36 Bratislava, Slovakia

**Keywords:** *Candida albicans*, *Staphylococcus aureus*, photodynamic inactivation, biofilm, polymer nanocomposites

## Abstract

*Candida albicans* and *Staphylococcus aureus* are opportunistic pathogens that cause life-threatening infections. This study focused on using photodynamic inactivation (PDI) to eliminate mixed biofilms of *C. albicans–S. aureus* formed on poly (urethane) (PU) discs functionalized with a nanocomposite layer containing phloxine B (PhB). Additionally, the effect of PDI on the *ALS3* and *HWP1* genes of *C. albicans* was examined in mixed biofilms. Spectral analysis showed a continuous release of PhB from the nanocomposite in Mueller–Hinton broth within 48 h, with a released amount of PhB < 5% of the total amount. The anti-biofilm effectiveness of the light-activated nanocomposite with PhB showed a reduction in the survival rate of biofilm cells to 0.35% and 31.79% for *S. aureus* and *C. albicans*, respectively, compared to the control biofilm on PU alone. Scanning electron microscopy images showed that the nanocomposite effectively reduced the colonization and growth of the mixed biofilm. While PDI reduced the regulation of the *ALS3* gene, the *HWP1* gene was upregulated. Nevertheless, the cell survival of the *C. albicans*–*S. aureus* biofilm was significantly reduced, showing great potential for the elimination of mixed biofilms.

## 1. Introduction

*Candida albicans* is currently one microorganism colonizing the human body, and under certain conditions, it becomes pathogenic and causes various infections from less dangerous oral aphthae and vulvovaginitis to life-threatening bloodstream infections [1,2,3]. Given the diversity of microbial species in the human microbiota, interactions occur within the same species and also between different species, genera, and kingdoms. Further, 30% to 60% of *C. albicans* infections are polymicrobial [4,5]. The most common co-infection is with the bacterium *Staphylococcus aureus* [4,6]. *C. albicans* can colonize various surfaces, on which it forms a biofilm [7]. Specific ALSs (agglutinin-like sequences) are formed in individual phases of biofilm development. Within the ALS family, Als3p is a cell surface protein of *C. albicans* that plays a key role in adhesion and biofilm formation and thus contributes to pathogenesis. Als3p has also been identified as a critical adhesin involved in the interactions between *C. albicans* and *S. aureus* [8,9]. It is encoded by the *ALS3* gene, mainly expressed by hyphae and pseudohyphae [10,11,12]. During biofilm growth, another protein, Hwp1 (hyphal wall protein), contributes to cell adhesion and subsequent hyphal formation [13,14]. The mycelial form of *C. albicans* provides a perfect base for colonization by other microorganisms, including *S. aureus*.

Biofilms are highly resistant to a broad spectrum of antimicrobial agents [15,16,17]. In addition to conventional options, alternative approaches to combat biofilm resistance have also been considered [18,19,20]. Numerous studies have demonstrated the benefit of photodynamic inactivation (PDI) in the eradication of biofilms formed by *S. aureus* [21,22,23,24,25,26] and *C. albicans* [22,27,28,29]. The reaction occurs through the interaction of the cells with a photosensitiser (PS) and subsequent irradiation with light of the appropriate wavelength. As a result of the interaction of PS with molecular oxygen, reactive oxygen species (ROS) are formed in a type I reaction with the production of oxidative radicals such as hydroxyl (OH˙), superoxide anions (O_2_˙^−^) and hydrogen peroxide (H_2_O_2_), and in a type II reaction with the formation of singlet oxygen (^1^O_2_). These ROS cause destruction that leads to cell death [30,31].

In this work, phloxine B (PhB) (2′,4′,5′,7′-tetrabromo-3,4,5,6-tetrachlorofluorescein sodium salt), which belongs to the xanthene dyes, was used as a PS. PhB is a commercially available dye commonly used in the cosmetic, pharmaceutical, and food industries, and it has optimal photoactive properties with visible light absorption at 540 nm [32,33]. As previously described, PhB was embedded on the surface of poly (urethane) (PU) discs in a thin film based on organically modified particles of layered silicate saponite (Sap) with a cationic surfactant (octadecyltrimethylammonium, ODTMA). The modification with the surfactant serves to achieve compatibility with PU and to increase the uptake of PhB in a thin film of the nanocomposite with the polymer chains fused with the Sap phase. This strategy leads to a higher antimicrobial efficacy of PS after PDI. It is supposed that the potential release could also contribute to the overall antimicrobial effect [21,23,33]. The continuous and controlled release of antimicrobial agents is a highly desirable strategy for treating infections, as well as for food packaging [34,35].

Based on previous work [21,23,24,33], the main objective of this work was to gain new information on how PDI affects the mixed biofilm of *C. albicans–S. aureus* that forms on a PU-modified surface. The important question was how the kinetics of PhB release affects the biofilm formed on the nanocomposite surface. It was also interesting to find out how PDI affects the expression of the *ASL3* and *HWP1* genes, as both genes are mainly expressed in the mycelial form of *C. albicans*, which is important for biofilm development.

## 2. Materials and Methods

### 2.1. Characterization of Microorganisms

The strains of yeast *C. albicans* SC 5314 [36] and the bacterium *S. aureus* CCM 3953 (corresponding to ATCC 25923, Czech Collection of Microorganisms, Brno, Czech Republic) were used in the experiments. Both strains were preserved at −80 °C in 1 mL of yeast extract peptone dextrose broth (YPD, Biolife, Milan, Italy) for *C. albicans* SC 5314 and Mueller–Hinton broth (MHB, Biolife, Milan, Italy) for *S. aureus* CCM 3953 supplemented with 30% glycerol (Centralchem, Bratislava, Slovakia). Before use, 50 μL of stock from microorganisms were inoculated into 50 mL of MHB or YPD and cultivated overnight on a shaker (Orbital Shaker-Incubator ES-20, BioSan, Riga, Latvia) at 150 rpm, 37 °C for 16 h.

### 2.2. Preparation of Functionalized Organoclay Films and Composites with Polymer

The stock solution of PhB was prepared according to the procedure described in the protocol of Dadi et al. (2021) [33]. Briefly, PhB was dissolved in distilled water, using a UV-Vis spectrophotometer (Visible Spectrophotometer S-220 UV, Boeco, Hamburg, Germany), and the absorbance of the solution at *λ* = 538 nm was measured. The antimicrobial effect of different concentrations of PhB (0.05; 0.1, and 0.5 mM) was tested in experiments.

The nanocomposite blocks were prepared according to the protocol Dadi et al. (2021) [21]. Briefly, the surface of PU (VARNISH-PU 2 KW, Isomat S.A., Thessaloniki, Greece) was modified with a thin layer based on a synthetic trioctahedral layered silicate—Sap, commercial name Sumecton (Kunimine Industries Co., Ltd., Tokyo, Japan)—that was used as an inorganic carrier of PhB. A cationic surfactant, ODTMA (octadecyltrimethylammonium; Sigma-Aldrich, Hamburg, Germany) was used to modify the Sap particles to prepare organoclay (OC) with efficient uptake of PhB and compatibility with the polymer. A thin film of organoclay with PhB (OC/PhB) was prepared by vacuum filtration colloid solution through Teflon filters (0.1 μm Omnipore, hydrophilic polytetrafluoroethylene—PTFE, 47 mm diameter, Millipore, Merck, Darmstadt, Germany). The PU composite with PhB (PUC/PhB) was obtained by fusing a liquid mixture of PU precursors poured onto the OC/PhB film. The optimum loading of PhB was achieved by the following component ratios: *n*_ODTMA_/*m*_Sap_ = 0.8 mmol⋅g^−1^ and *n*_PhB_/*m*_Sap_ = 0.21 mmol⋅g^−1^.

### 2.3. Phloxine B Release from Composite

The kinetics of drug release was tested using the following time points: 2, 4, 8, 15, 30, 60 (min), 2, 4, 6, 8, 24, 48 (h). The PU discs were immersed in 50 mL of MHB, and 1 mL of the medium was withdrawn at each time point and used for analysis. The amount of PhB released was calculated based on the concentrations in the medium determined at each time point, plus the amounts in the previously collected 1 mL samples. To determine the concentrations of released dye, PhB standards were prepared in the same medium with different concentrations: 2.40, 0.240, 0.024, 4.69, 2.34, 1.17, 0.586, 0.293, 3.84, 0.768, 0.154, 0.031 μmol L^−1^. The standards were stable, and no change in PhB concentration was observed over time. The spectra were measured using an Agilent 8453 UV/VIS Spectrophotometer (Agilent, Santa Clara, CA, USA) in the visible spectral range at RT. Since the medium caused significant light scattering and nonlinear baseline curvature due to the presence of macromolecular substances, a chemometric decomposition of the spectral matrix (Unscrambler^®^ 10.3, Camo, Oslo, Norway) was performed to obtain pure PhB spectral profiles. The decomposition was performed for both the samples and the standards. Using the multilinear curve resolution–alternating least squares (MCR), a pure PhB spectral profile was obtained without interference from other substances. The pure PhB spectra could be obtained by multiplying the spectral component corresponding to PhB by the values of arbitrary concentrations obtained by MCR. The arbitrary concentrations obtained by MCR were used directly to determine the concentrations of released PhB. For standards with known PhB concentrations, a linear regression based on the Lambert–Beer law was performed (Origin Pro 2023b, Origin Lab Corporation, Northampton, MA, USA). Despite light scattering by the medium, the data followed a nearly perfect linear trend (*R*^2^ = 0.993) after chemometric data analysis. The amount of PhB released over time was calculated from the determined concentrations. The results were analyzed using the Weibull function model expressed by Equation (1):

(1) $$a(t)=a_{\infty }{(1-e}^{{-(kt)}^{d}})$$

*a*(*t*) is the function of PhB release with time, $a_{\infty }$ is the maximum fraction released, *k* is a rate constant, and *d* is a Weibull shape parameter controlling curve shape.

### 2.4. Spectral Characterization of the Film and the Composites

The UV-Vis spectra of OC/PhB and PUC/PhB were measured using a Shimadzu 2600i spectrophotometer equipped with an integrating sphere (ISR-2600Plus) for reflectance measurements (Shimadzu, Kyoto, Japan). The measurements were performed in diffuse reflectance mode at laboratory temperature. An Omnipore PTFE filter (Merck Millipore, Burlington, MA, USA) was used as a blank to record the baseline and also served as support for the measured samples. The samples had a square shape with a size of 1 cm. The spectra were evaluated as −log*R*, which semi-quantitatively reflects the amount of absorbed light and chromophore concentration. Fluorescence spectra, including excitation–emission matrices (EEMs), were recorded using a Duetta spectrophotometer controlled by EZSpec software, version 1.4.3.3 (Horiba, Burlington, ON, Canada). The measurement conditions were kept the same to compare both the emission profiles and the intensities of the samples.

### 2.5. Formation of 24 h Dual Biofilm

The preparation of a mixed biofilm has already been described in the protocol of Kendra et al. (2024) [20]. Briefly, an overnight culture of *C. albicans* was harvested by centrifugation (Universal 32 R Hettich Zentrifugen, Tuttlingen, Germany), followed by washing with phosphate-buffer saline (PBS, 137 mM NaCl, 2.7 mM KCl, 10 mM Na_2_HPO_4_, 2 mM KH_2_PO_4_, pH 7.4, all chemicals from Centralchem, Bratislava, Slovakia) and centrifugation. The pellet was then resuspended in PBS, and the final density of *C. albicans* was adjusted to 4 × 10^6^ cells/mL in MHB supplemented with 2% D-glucose (Centralchem, Bratislava, Slovakia).

The suspension of *S. aureus* was prepared from an overnight culture. After 16 h, the culture was transferred into fresh MHB supplemented with 2% D-glucose and adjusted to an optical density (OD) of OD_570_ = 0.05 and incubated at 150 RPM (Thermostatic cabinet, Lovibonds, Biosan, Riga, Latvia), 37 °C until the exponential phase (OD_570_ = 0.5; ≈2.5 h), corresponding to ≈1 × 10^8^ cells/mL.

The dual biofilm of *C. albicans–S. aureus* was prepared as follows: 200 μL or 1 mL of *C. albicans* suspension in MHB supplemented with 2% D-glucose was added into a 96- and 24-well microtiter plate (Sardstedt AG & Co., Nümbrecht, Germany), respectively, and incubated at 37 °C (Thermostatic Cabinet, Lovibond, Dortmund, Germany) for 7 h. Then, 50 μL or 250 μL *S. aureus* suspension was added to the *C. albicans* biofilm. Cultivation continued for a further 17 h to obtain the 24 h biofilm.

### 2.6. PDI of Dual Biofilm C. albicans–S. aureus

The effectiveness of PDI was tested on 24 h dual biofilms, prepared as described in Section 2.5 according to the procedure of Kendra et al. (2024) [20] and Bugyna et al. (2024) [24] with some modifications. Briefly, the medium was removed from the 24 h dual biofilm, and biofilms were then incubated for 2 h in the dark at room temperature (RT) with 100 µL of a 0.05, 0.1, or 0.5 mM PhB. The samples were irradiated with a green laser (*λ* = 532 nm, 100 mW, Alligator, MZTech s.r.o., Košice, Slovakia) for 300 s. After irradiation, the biofilm layers were scraped off and serially diluted in PBS. Dilutions were plated onto MHA plates with fluconazole (4 µg/mL, Zentiva, Prague, Czech Republic) and YPD with gentamicin (4 µg/mL, Applichem, Darmstadt, Germany), and then cultivated at 37 °C for 24 h. The results were evaluated by counting the colony-forming units (CFU), which were determined as the percentage of viable cells treated with PhB without PDI and the cells treated with PDI in the dark compared to the control sample (100%) without irradiation. Experiments were performed in three parallel wells and were repeated in at least three independent replicas.

### 2.7. PDI of Biofilms Formed on Nanocomposite

The anti-biofilm activity was tested according to the procedure of Dadi et al. (2021) [21] and Bugyna et al. (2024) [24] on PU discs alone that represented the control, on PUC, and on PUC modified with hybrid film OC/PhB, with and without irradiation (PDI). All types of PU discs were cut into 1 cm × 1 cm squares, sterilized by UV irradiation on both sides for 10 min, and fixed on the bottom of a 24-well microtiter plate containing 150 µL of 2% agarose (Sigma Aldrich, Darmstadt, Germany). The biofilms were prepared as described in Section 2.5.

After 24 h, the medium was carefully removed and the biofilms that had formed on the PUC/PhB discs were irradiated with a green laser for 300 s. Then, all disc samples were transferred into 5 mL Eppendorf tubes with 2 mL PBS (one per each), sonicated at 55 kHz (Branson 200 ultrasonic cleaner, Danbury, CT, USA), vortexed for 5 min (Multi-Vortex V-32, Biosan, Riga, Latvia), and serially diluted in PBS. The dilutions were plated onto MHA plates with fluconazole or YPD plates with gentamicin and cultivated at 37 °C for 24 h. Then, CFUs per 1 mL were calculated. Experiments were prepared in three parallel wells and repeated in at least three independent replicas. The results represent cell survival compared to control growth on PU alone (100%) with standard deviations.

### 2.8. Scanning Electron Microscopy

A 24 h dual biofilm of *C. albicans–S. aureus* was prepared on all types of material as previously described in Section 2.5. Samples were prepared for scanning electron microscopy (SEM) according to the protocol of Radochová et al. (2023) [37]. Briefly, blocks with biofilms were transferred into a 24-well plate, washed with 1 mL PBS, and fixed for 1 h in the dark with 4% paraformaldehyde (Sigma-Aldrich, Steinheim, Germany) at RT. After incubation, the fixative solution was removed, the samples were washed twice in PBS for 10 min, and then post-fixed with 1% osmium tetroxide (EMS, Hatfield, PA, USA) supplemented in PBS for 1 h in the dark at RT. Then, the samples were washed twice in PBS and once in deionized water for 10 min at RT. Dehydration was performed by washing the samples for 15 min at RT in serial dilutions of 25%, 50%, 70%, 95% ethanol/distilled water solutions and 100% ethanol (Sigma-Aldrich, Steinheim, Germany) at RT for 30 min. The samples were dried in the dark at RT, sputter-coated with carbon (20 nm) by Sputter Coater QISOT ES (Quorum Technologies, Lewes, UK), and then fixed with a carbon tape specific to the sample holder of the SEM. The samples were finally analyzed under a JSM-7100F microscope (JEOL, Tokyo, Japan).

### 2.9. Isolation of RNA from Nanocomposites and Reverse Transcription to cDNA

RNA isolation from dual biofilms on antimicrobial surfaces was performed at two different time points—after 10 h and 24 h of biofilm development in 24-well plates (preparation of biofilm was described in Section 2.5). To obtain a sufficient amount of RNA from the samples, 25 pieces of 1 cm × 1 cm squares from each type of material were collected into a 50 mL Falcon tube with 15 mL of PBS. All samples were sonicated at 55 kHz for 5 min, vortexed for 5 min, and centrifuged at 5000× *g*, 5 min, 15 °C. The supernatant was removed along with the nanocomposites, and the cells were transferred to a 2 mL microcentrifuge tube. RNA isolation was processed by using a GeneJET RNA Purification Kit (Thermo Scientific, Waltham, MA, USA) with the subsequent modifications: 200 μL of yeast lysis (containing β-mercaptoethanol and 200 U of lyticase) was added to the pellet of each sample. The pellet was vortexed for 1 min and incubated in the lysis solution at 30 °C for 60 min. In the next step, 300 μL of yeast lysis buffer and 200 µL of sterile glass beads (Glass Beads, Acid-Washed ≤ 106 µm, Sigma Aldrich, Darmstadt, Germany) were added to the samples and vortexed for 60 min at RT (2550 RPM, Vortex—Genie 2, Scientific Industries, Bohemia, NY, USA). Then, the protocol was followed according to the instructions of the kit’s provider.

The RNA isolated in this way was subsequently purified with DNase I, RNase-free (Thermo Scientific, Waltham, MA, USA) and the samples were stored at −80 °C or used immediately for transcription to cDNA using a Maxima First Strand cDNA Synthesis Kit for Real-time PCR (RT-PCR, Maxima First Strand cDNA Synthesis Kit, Thermo Scientific, Waltham, MA, USA). The synthesized cDNA was stored at −20 °C or used immediately in real-time PCR (RT-qPCR).

### 2.10. Expression of the ALS3 and HWP1 Genes in C. albicans

The primers for the *ACT1*, *ALS3,* and *HWP1* genes were obtained from previous studies [38,39]. The thermal programme for the three-step RT-PCR was set to 1 cycle for initial denaturation at 95 °C for 12 min; 40 cycles of denaturation at 95 °C for 0:15 min; annealing at 60 °C for 1:00 min; 1 cycle of complementary phase at 95 °C for 0:15 min; 60 °C for 0:15 min; and 95 °C for 0:15 min. The reaction was performed using 5× HOT FIREPol ^®^ EvaGreen^®^ qPCR Mix Plus (Solis BioDyne, Tartu, Estonia), an Mx3000P qPCR instrument (Agilent Technologies, Inc., Santa Clara, CA, USA), and data analysis was performed using Stratagene MxPro software V.4.10 from Agilent Technologies. The relative change in gene expression was determined according to the 2^−ΔΔC_T_^ method of Livak et al. (2001) [40]. The 10- and 24 h *C. albicans*–*S. aureus* biofilms isolated from PU alone served as a control set and were normalized to 1. The expression of the two genes *ALS3* and *HWP1* was normalized to that of the housekeeping gene *ACT1*. The experiments were performed in triplicate from three parallel wells from two mutually different isolates.

### 2.11. Statistical Analysis

The normality of the data was determined by the Shapiro–Wilk test. For further analysis of the effectiveness of PDI, the Kruskal–Wallis nonparametric test was used. Dunn’s comparison test was used to determine significant differences between the groups. Analysis of relative gene expression was completed with Welch’s ANOVA in GraphPad Prism software v. 9 (Graph Pad, San Diego, CA, USA). Differences were considered significant at various *p*-values: *p* < 0.05 (*), *p* < 0.01 (**), *p* < 0.001 (***), and *p* < 0.0001 (****).

## 3. Results

### 3.1. Kinetics of PhB Release from Composite

**Figure 1:**
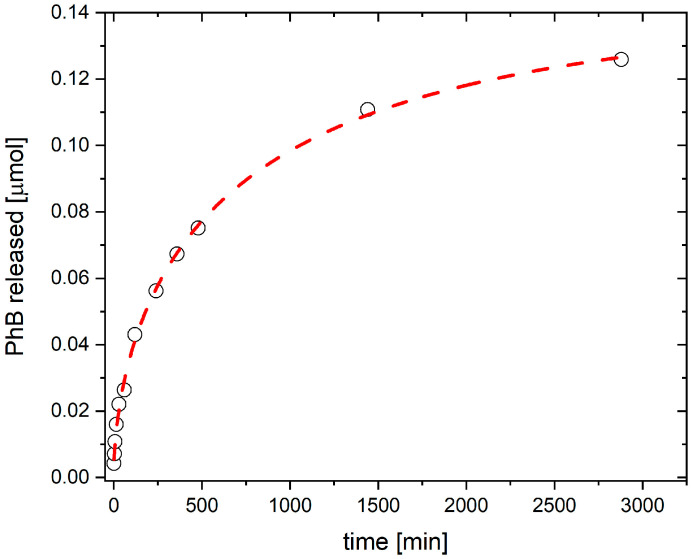
The release of PhB from the PUC/PhB composite disc over time. The red dashed line shows the profile of the Weibull model function (Equation (1)). The values of the parameters and the statistical relevance of the fit are summarized in Table 1.

The PUC/PhB nanocomposite was prepared with 3.125 μmol PhB, according to the protocol of Dadi et al. (2021) [21]. The dye was concentrated in the thin film of the composite on the surface of the PU disc. Figure 1 shows the kinetics of PhB release from the disc in the MHB medium within 48 h. The PhB amounts showed that <5% of the total amount was released. The PhB release could not be fitted with a simple exponential growth but is well described by a Weibull model function (Equation (1)). This model provided an excellent fit to the data, indicating minimal residual error and excellent explanatory power (Table 1). The Weibull shape parameter *d* = 0.57 shows a parabolic release profile with a high initial release rate followed by a decreasing rate over time, typical for systems with a surface-adsorbed drug and diffusion-limited release from a composite matrix. The burst release was observed in the first few hours, reaching a half-life of about 7.4 h. According to the model, about 75% of the maximal amount $a_{\infty }$ was released during the first 24 h. Between 24 and 48 h, about 13% of the limiting amount $a_{\infty }$ was released, and a similar amount was expected for the rest of the release by extrapolating the function (Equation (1)) until the maximum value ($a_{\infty }$) is reached.

### 3.2. Spectral Characterization of the Film on Membrane and Nanocomposites

Reflectance and fluorescence spectra were measured and are shown in the Supplementary Data (SD) File (Figures S1, S2A,B and S3A–C). Figure S1 summarizes the UV-vis spectra of the film on the membrane and the composite before and after PhB release. They were compared with the blank of the PU disc alone. Since the dye contents were very high, the spectra provided only qualitative information (see discussion and some comments in the SD file). Fluorescence spectroscopy provided greater sensitivity in detecting differences between the samples (Figure S2A,B). The solid samples of the film on the membrane, and the composite before and after PhB release, were compared. The spectra obtained by excitation at 520 nm (Figure S2A) and 470 nm (Figure S2B) showed some differences. When comparing the emission spectra of the OC/PhB film and the PUC/PhB composites, a significant reduction in fluorescence intensity was observed when PU was involved. After the release of the dye, a further decrease in emission occurred. The differences in emission intensity between the film and the composite samples were much smaller when excited at 470 nm (Figure S2B). For a more detailed analysis of the fluorescence properties, EEMs were recorded for the film and the composites before and after dye release. The 3D spectra of EEMs provide a comprehensive overview of the differences in the fluorescence behaviour of the samples (Figure S3A–C). The OC/PhB film exhibited the highest fluorescence intensity (Figure S3A), as shown by the intensity scale reaching up to 4 a.u. (arbitrary unit). Excitation was most efficient at longer wavelengths, with efficiency decreasing significantly at shorter wavelengths. In contrast, the PUC/PhB composites showed significantly reduced fluorescence, with intensities as low as 0.7 a.u. (Figure S3B). In this range, excitation at shorter wavelengths, such as 470 nm, became more apparent. However, the most efficient excitation for this sample was still in the 520–530 nm range. Another change occurred after the dye was released from the composite by the MHB medium (Figure S3C). Although the overall intensity scale remained almost the same (0.8 a.u.), the excitation wavelength corresponding to the emission maximum shifted significantly. The emission attributed to the monomeric form (excitation at 520–530 nm), which dominated in the OC/PhB film and the PUC/PhB composite before dye release (Figure S3A,B), disappeared. The excitation spectrum in Figure S3C showed a clear maximum at 460–480 nm.

### 3.3. Effectiveness of PhB After PDI

In previous experiments by Dadi et al. (2021) [33], the efficacy of PhB on biofilms of the bacterium *S. aureus* was tested. In this work, the effect of PhB against the biofilm of *C. albicans* was determined. Therefore, different concentrations of PhB (1, 0.5, 0.1, 0.05 mM) were tested in preliminary experiments to determine the inhibitory effect on the 24 h biofilms of *C. albicans*. The results showed that a higher concentration of PhB was most effective, with the survival of biofilm cells irradiated for 120 s being approximately 16% compared to the control without PhB. However, this concentration also showed an antimicrobial effect in the dark, which is why it was not used for further experiments (SD, Figure S4).

**Figure 2:**
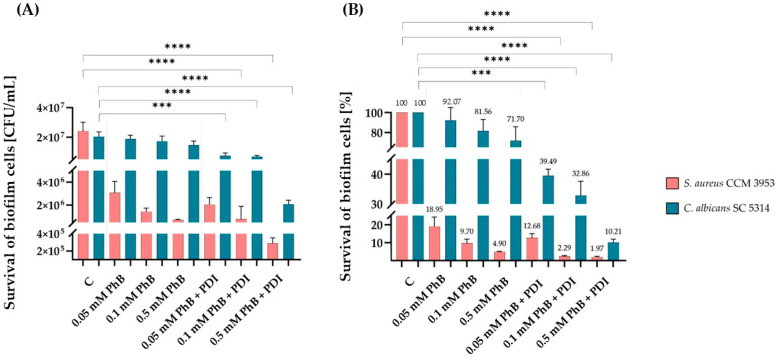
Determination of the inhibitory effect of PDI in the presence of PhB on a 24 h dual biofilm of *C. albicans* SC5314 and *S. aureus* CCM 3953, shown as CFU/mL (**A**); percentage of surviving cells before and after PDI (300 s) (**B**). C represents samples without irradiation; PhB represents toxicity control without PDI; and PhB + PDI is the sample after irradiation. The results were considered to be significant at different *p*-values: *p* < 0.001 (***) and *p* < 0.0001 (****).

The lower concentrations, 0.5, 0.1, and 0.05 mM PhB, were then tested on the 24 h dual *C. albicans–S. aureus* biofilms, and the irradiation was extended to 300 s (Figure 2A,B). The results showed that the survival of the biofilm cells was significantly reduced. The highest inhibition was observed at 0.5 mM PhB, with a reduction from 2.4 × 10^7^ CFU/mL to 2.8 × 10^5^ CFU/mL of *S. aureus*, corresponding to approximately 1.97% surviving cells after irradiation. For *C. albicans*, this was from 2.03 × 10^7^ to 2.07 × 10^6^ CFU/mL after PDI, with only 10.21% of cells surviving after PDI compared to the control samples without irradiation.

**Figure 3:**
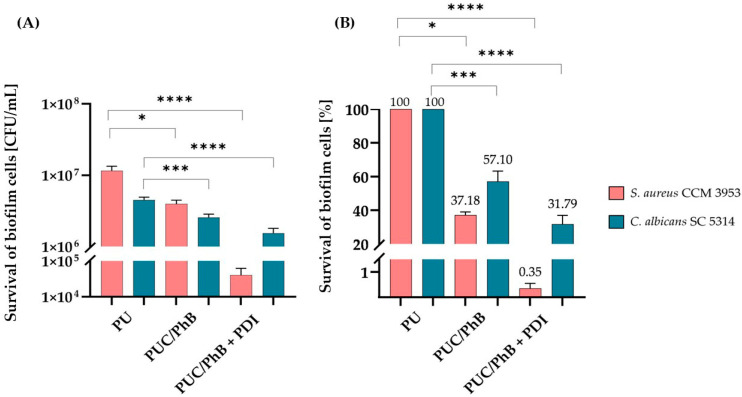
Determination of the inhibitory effect of nanocomposite on a 24 h dual biofilm of *C. albicans* SC5314 and *S. aureus* CCM 3953 shown as CFU/mL (**A**); percentage of surviving cells before and after PDI (300 s) (**B**). PU represents the control samples without irradiation, PUC/PhB represents the toxicity control without PDI, and PUC/PhB + PDI is the sample after irradiation. The results were considered to be significant at different *p*-values: *p* < 0.05 (*), *p* < 0.001 (***), and *p* < 0.0001 (****).

**Figure 4:**
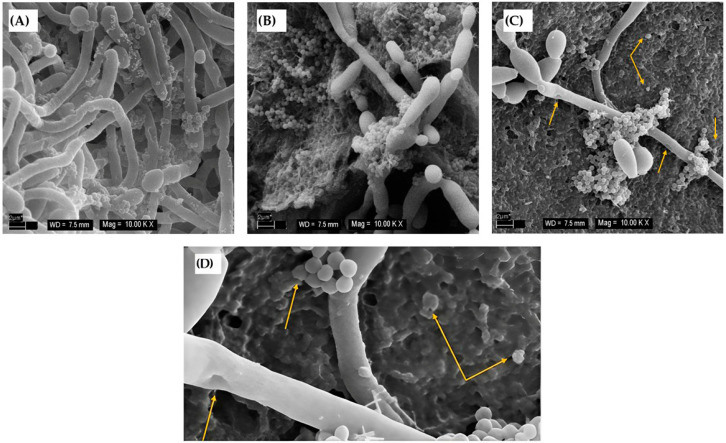
Determination of the inhibitory effect of the nanocomposite on a 24 h dual biofilm of *C. albicans* SC5314 and *S. aureus* CCM 3953; (**A**) the control biofilm on PU; (**B**) biofilm on PUC/PhB represents the dark-toxicity control; (**C**) the sample PUC/PhB + PDI for 300 s; (**D**) details of the damaged yeast and bacteria.

The anti-biofilm effectiveness of the light-activated nanocomposite with immobilized PhB compared to the control PU alone is summarized in Figure 3A,B. The results showed that the nanocomposite without PDI also exhibited significant anti-biofilm activity, but after irradiation, the survival rate of biofilm cells was even more reduced; from 1.1 × 10^7^ CFU/mL to 4 × 10^4^ CFU/mL corresponding to a minimal survival rate (0.35%), and from 4.5 × 10^6^ to 1.5 × 10^6^ CFU/mL, corresponding to 31.79% for *S. aureus* and *C. albicans*, respectively. The results were also supported by SEM images. The photo in Figure 4A shows the adhesion and mutual interaction between *C. albicans* and *S. aureus*, with cells forming a robust biofilm on PU, which serves as a control. The PUC/PhB film (Figure 4B) showed a significant inhibition of the growth of both yeast and bacterial cells. In addition, a reduction in the development of *C. albicans* hyphae was also proven, confirming the anti-biofilm activity of the composite without irradiation. After irradiation, the growth of both microorganisms, but mainly *S. aureus*, was reduced on the PUC/PhB discs (Figure 4C); further details in the image (Figure 4D) show the disruption of *C. albicans* and *S. aureus*.

### 3.4. Relative Change in Expression of the ALS3 and HWP1 Genes

**Figure 5:**
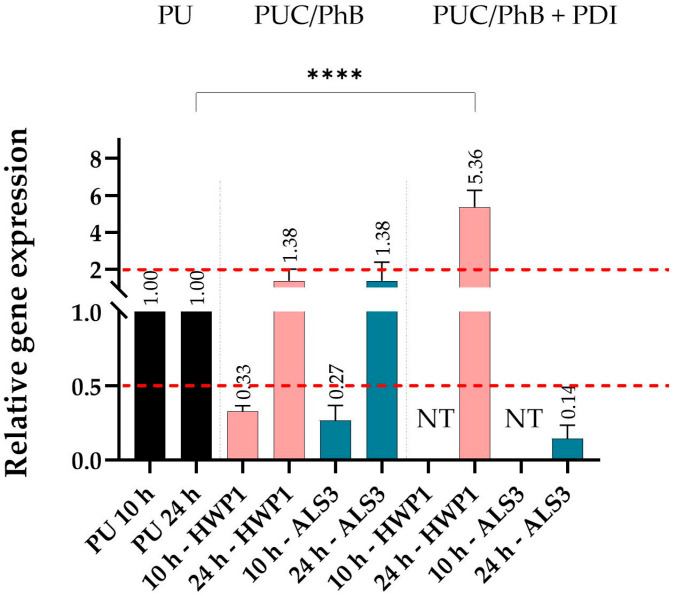
Relative changes in expression of the *ALS3* and *HWP1* genes of *C. albicans* in a mixed 24 h biofilm of *C. albicans–S. aureus*; samples on PU after 10 or 24 h were controls and set to 1; ACT1 was used as the housekeeping gene. NT—not tested. A *p* < 0.0001 (****) was considered extremely highly significant.

This experiment was conducted at two time points, after 10 and 24 h. The shorter point was chosen because the expression of the *ALS3* gene is particularly important at the beginning of biofilm formation, and also to learn about the possible effect of PhB release. The *HWP1* gene can be expressed in the later phase and is more associated with the development of hyphae. However, after irradiation of the 10 h samples, the mass of biofilm cells was too low to obtain enough RNA for RT-PCR. Therefore, these samples were not tested in RT-PCR (Figure 5, NT). The gene expression could only be determined for a 24 h biofilm. The results of RT-qPCR showed that the relative expression of both *ALS3* and *HWP1* genes decreased in the 10 h biofilm without irradiation compared to the control biofilm on PU alone, while in the 24 h biofilms, the expression of both genes increased only slightly. After irradiation, the expression of the *ALS3* gene was even lower compared to the non-irradiated sample, in contrast to the *HWP1* gene, which was significantly upregulated compared to the control and even the non-irradiated sample (Figure 5).

## 4. Discussion

Currently, *C. albicans* and *S. aureus* are among the most significant pathogens causing bloodstream infections, resulting in substantial morbidity and mortality in hospitalized and immunocompromised patients [41,42,43]. Although yeast biofilms represent one of the major medical problems, research is mainly focused on controlling bacteria [44,45]. Additionally, the issue of polymicrobial biofilms remains poorly understood [43]. Many papers have already described the antimicrobial efficacy of PDI against *C. albicans* or *S. aureus* biofilms [31,46,47], but the testing of mixed biofilms of both microorganisms has not been published frequently [20,48,49].

The photoactive nanomaterial could be a strategy to prevent or control microbial biofilms. In the case of the photoactive nanocomposite presented in this paper—xanthene dye—PhB was used as part of a PU-based functionalized composite. The effect of this nanocomposite against *S. aureus* has already been described [21,23,33], but its effectiveness on mixed *C. albicans*–*S. aureus* biofilms has not yet been investigated. In addition, this work was also devoted to a deeper study of the kinetics of PhB release from the nanocomposite, as this phenomenon is crucial for the overall activity. Presumably, some substances in the MHB medium could be absorbed on the surface and in the composite pores, leading to the exchange and gradual release of the PhB molecules. The release of PhB from the surface of the composite, as described by the Weibull function, could influence the formation of biofilm growth at different stages due to its characteristic kinetic profile. It has been shown that release in the first hours of biofilm development could reduce the ability of microorganisms to colonize the nanocomposite and even kill them, resulting in the formation of small, individual “islands” of bacterial clusters and hyphae compared to a fully developed mature biofilm on PU without PhB. Less than 5% of the total amount of PhB present in the disc was released. A similar nanocomposite with half the amount of PhB in the sample (0.1 mmol g^−1^) released less than 1.5% [21]. This work has shown that saturation of the nanocomposite with PhB to the maximum limit (0.21 mmol g^−1^) is important to boost the release of a significantly larger amount of PS and thus increase the anti-biofilm efficiency. On the other hand, the half-life of the release process (7.4 h) is not so different from the 6 h observed in a previous study [21]. A large PhB release and the PDI of a 10 h biofilm led to a decrease in the number of viable microorganisms and thus to a very low RNA yield. This was also reason that the mature (24 h) biofilm was tested. Moreover, mature biofilm represents a phase of biofilm development that is the most difficult to eradicate [50,51,52]. Considering the release kinetics, it is logical that after 10 h, only very few microorganisms have adhered. Therefore, several discs were used for RNA isolation (15 PUC/PhB vs. 25 PUC/PhB + PDI). Even with this number, the amount of isolated RNA was too low for RT-PCR. SEM images showed that the PUC/PhB were also effective against a 24 h biofilm, and even after PDI, the cells of *C. albicans* and *S. aureus* were disrupted.

Spectral analysis of the materials (film and nanocomposites) helped to determine the changes in dye release. The materials retained dye in the form of molecular aggregates. Band broadening in the UV-Vis spectra of OC/PhB and PUC/PhB confirmed a high concentration of chromophores and indicated the prevalence of molecular aggregates in the solids. Significant differences were observed between the film and the PU composites, while the spectra of the composite before and after dye release were almost identical. This confirms that only a small fraction of the dye was released. The presence of dye aggregates was confirmed by emission spectra. When comparing the OC/PhB film and the PUC/PhB composites, a significant reduction in fluorescence intensity was observed for the PUC/PhB composites. In the film on the membrane, the dye was located in the lipophilic environment of the alkyl chains of the ODTMA surfactant, preserving some molecules in a photoactive form. Specific interactions between the PU chains and the dye molecules in the composites led to reduced emission. Interpretations include the formation of aggregates and fluorescence quenching or the reaction/decomposition of a fraction of PhB molecules. However, the reaction was not confirmed by spectral analysis of the released PhB in MHB. The 3D EEM spectra of the film on the membrane and the composites provided a comprehensive view of the differences in the fluorescence behaviour of the samples. The OC/PhB film exhibited the highest fluorescence intensity. The high emission from the film can be attributed to a relatively large amount of unaggregated dye molecules, as shown in the results described above. In contrast, the PUC/PhB composites showed significantly reduced fluorescence. A surprising change occurred in the spectrum of the PUC/PhB composite after the release of a part of PhB into MHB. The excitation wavelength corresponding to the emission maximum of PhB was significantly shifted to lower wavelengths. These changes can be interpreted as a gradual and preferential release of monomeric PhB molecules from the composite. On the other hand, the dye molecules, which form molecular aggregates, remained fixed in the polymer composite and could not be washed out by the substances present in MHB.

These data confirm that the nanocomposite was still active after 24 h. The activity has to be significantly higher against *S. aureus* bacteria, which has already been demonstrated in previous works [21,23,33]. Based on the mentioned results obtained with a single *S. aureus* biofilm, it is evident that PDI was more effective compared to the effect against *S. aureus* in mixed biofilm. A higher anti-biofilm effectiveness of PDI was also observed in the single biofilm of *C. albicans* (Figure S5). The reduced efficacy of PDI in the case of mixed biofilm is likely due to the increased complexity of the extra polymeric matrices or altered microbial physiology in co-culturing. Moreover, in our experiment, irradiation was used only one time; therefore, the inhibition obtained (almost 100% and 68% inhibition for *S. aureus* and *C. albicans*, respectively) is a very promising result. In clinical practice, the duration of the irradiation period can be adjusted according to the application, and usually, it is repeated several times in order to achieve the best possible anti-microbial effect [53,54,55]. The goal of the presented work was to show that the material is active against mixed biofilm, and that it works under determined conditions. Since part of the study also included experiments focused on gene expression, it was necessary to find a balance between biofilm reduction and a minimal yield of RNA isolated from irradiated biofilm. In the yeast *C. albicans*, the effect of PDI on survival was lower compared to bacteria in mixed biofilm, but the expressions of *ALS3* and *HWP1* genes coding for proteins important for biofilm and hyphal development were significantly affected. However, this statistical significance has not been labelled, as values of nearly 2-fold exchange are not relevant from a molecular point of view. On the other hand, despite the exchange regulation below 0.5 for some genes, which is considered to be significant, this was also not marked, as the research was primarily interested in upregulated genes, as downregulation is a common phenomenon that can be observed under adverse conditions (the influence of antimicrobial compounds, lack of nutrients, etc.). The effect of PDI on both genes has already been investigated [56,57]. The aforementioned studies confirmed a significant reduction in the expression of *ALS1*, *HWP1*, *CAP1*, CAT1, and *SOD1* genes, as well as *ALS3*, *HWP1*, *BCR1*, *TEC1*, *CPH1*, and *EFG1* genes in the presence of curcumin (40 and 80 μM) and photodithiazine (100 and 200 mg/L), or methylene blue (300 μM) and erythrosine (400 μM), respectively [56,57]. The reduction in the expression of these genes suggests that PDI can reduce the virulence of *C. albicans*. In agreement with the aforementioned work, it was observed that the relative expression of the *ALS3* gene decreased in a 24 h biofilm after PDI. On the other hand, an upregulation of the *HWP1* gene was observed. This could be due to the fact that *C. albicans* tries to maintain homeostasis and survive in the mycelial form, which is better adapted to the stress response [58,59]. This finding correlates with a paper by Mohammed et al. (2024), which showed that exposure of *C. albicans* to oxidative stress stimulated the expression of the *MLH1*, *HWP1*, and *ERG11* genes, as part of the defence mechanism [60].

The presented results show that although *C. albicans* and *S. aureus* survive in a mixed biofilm, they respond independently to PDI, as evidenced by the much higher susceptibility of *S. aureus* to PDI despite the presence of the mycelial form of *C. albicans*. The higher susceptibility of *S. aureus* is probably related to its ability to respond to oxidative stress, which is much more complex in yeasts as representatives of eukaryotic cells.

## 5. Conclusions

This study showed the promising potential of PDI using PS PhB in PU-based nanocomposites for the control of mixed *C. albicans–S. aureus* biofilm. The main questions of this work were how the kinetics of PhB release affects the biofilm formed on the photoactive nanocomposite, and whether PDI affects the expression of *ASL3* and *HWP1* genes, as these are expressed in the mycelial form of *C. albicans*, which forms the basis for *S. aureus* attachment and growth. Spectral analyses showed that continuous PhB release affected the survival of biofilm cells and gene expression in *C. albicans*, not only in the first hours of biofilm formation but also after 24 h, when a fully developed biofilm had formed on the control PU discs. The effectiveness of PDI was higher against *S. aureus* than against *C. albicans*, which was to be expected, as yeasts have a more effective defence against oxidative stress. On the other hand, PDI affected the regulation of the two genes tested. While the *ALS3* gene was significantly downregulated after PDI, the expression of *HWP1* increased, which is probably related to the ability of the mycelial form of *C. albicans* to cope with the stress. Nevertheless, the mixed biofilm was significantly eradicated by the PDI, as shown in the SEM images. Nowadays, antimicrobial resistance is a global challenge, and PDI and nanocomposites with photoactive properties could provide an alternative to conventional treatment.

## Figures and Tables

**Table 1:** The parameter values of the Weibull model describing the release of PhB molecules from the PU composite disc.

Value	*t*-Value	Prob > |*t*|	Dependency
$a_{\infty }$	0.147 ± 0.006	25 × 10^−9^	0.96
*d*	0.57 ± 0.02	28 × 10^−10^	0.77
*k*/min^−1^	(11.9 ± 1.7) × 10^−4^	7 × 10^−5^	0.97

Statistical parameters: Reduced *χ*^2^: 2.68 × 10^−6^, residual sum of squares: 2.41 × 10^−5^, *R*^2^ = 0.9987, and adjusted *R*^2^ = 0.9984.

## Data Availability

The data presented in this study are available on request from the corresponding author due to some dates have not yet been published while others have been published. Some data presented in this study are available on https://zenodo.org/records/15497041 (accessed on 23 May 2025).

## Supplementary Materials

The following supporting information can be downloaded at https://www.mdpi.com/article/10.3390/jof11080582/s1, Figure S1: The UV-vis spectra of the OC/PhB film, PU, and PUC/PhB composite before and after the release of the dye. The spectra are shifted vertically. Figure S2: Emission spectra of the OC/PhB film, and its composite with PUC/PhB before and after the release of the dye. The excitation wavelength was 520 nm (A) and 470 nm (B). Figure S3: Excitation/Emission 3D plots of the OC/PhB film (A), and its composite with PU before (B) and after the release of the dye (C). Figure S4: Inhibitory effect of PDI on 24 h biofilm of *C. albicans* SC 5314 after application of different concentrations of PhB with irradiation for 120 s; C-control without PhB. The results were considered to be significant at different *p*-values: *p* < 0.01 (**) and *p* < 0.0001 (****). Figure S5: Inhibitory effect of nanocomposite on a 24 h single biofilm of *C. albicans* SC5314 shown as CFU/mL (A); and percentage of surviving cells before and after PDI (120 s) (B). PU represents the control samples without irradiation, PUC/PhB represents the toxicity control without PDI, and PUC/PhB + PDI is the sample after irradiation. The results were considered to be significant at different *p*-values: *p* < 0.05 (*), *p* < 0.001 (***), and *p* < 0.0001 (****).
