# Supplementary material for: Mixed Candida albicans–Staphylococcus aureus Biofilm Is Reduced by Light-Activated Nanocomposite with Phloxine B
Source: J Fungi (Basel). 2025 Aug 5;11(8):582. doi: 10.3390/jof11080582 (PMC12387639; doi:10.3390/jof11080582)
Supplement: Supplementary file 1 [file jof-11-00582-s001.zip › jof-3694140-supplementary.pdf]

Supplementary data

Mixed *Candida albicans–Staphylococcus aureus* Biofilm Is Reduced by Light-Activated Nanocomposite with Phloxine B

Jarmila Czucz Varga [1], Juraj Bujdák [2,3] and Helena Bujdáková [1,*]

[1] Department of Microbiology and Virology, Faculty of Natural Sciences, Comenius University in Brati-slava, Mlynská dolina, Ilkovičova 6, 842 15 Bratislava, Slovakia
[2] Department of Physical and Theoretical Chemistry, Faculty of Natural Sciences, Comenius University in Bratislava, Mlynská dolina, Ilkovičova 6, 842 15 Bratislava, Slovakia
[3] Institute of Inorganic Chemistry, Slovak Academy of Sciences, Dúbravská cesta 9, 845 36 Bratislava, Slovakia
* Correspondence: helena.bujdakova@uniba.sk; Tel.: +421-2-9014-9436

Spectral characterization

UV-Vis spectra (Figure S1) are expressed by the -log(*R*) function, where *R* represents reflectance, semiquantitatively related to the light absorption by the samples. Band broadening confirms complex spectral properties of the solids. The highest amount of molecular aggregates was confirmed in the composites with broad bands in the visible region. The enhanced intensity at lower wavelengths in the spectra of composites (~500 nm) confirmed the enhanced formation of molecular aggregates.

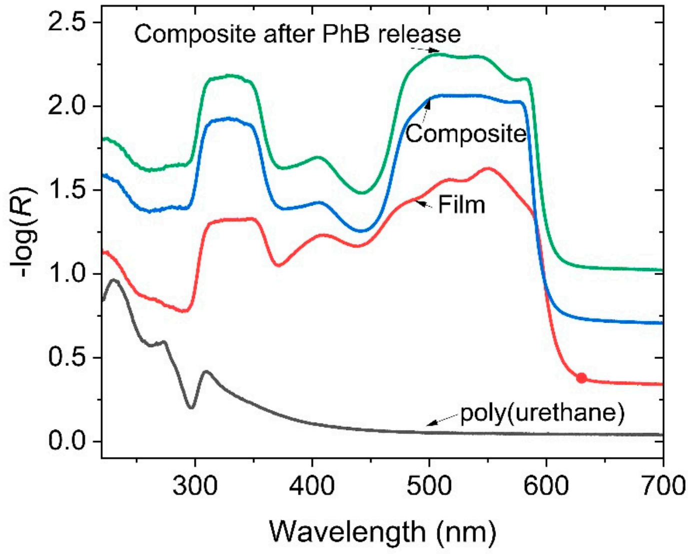

Figure S1. The UV-vis spectra of the OC/PhB film, PU, and PUC/PhB composite before and after the release of the dye. The spectra are shifted vertically.

A dilute PhB solution exhibited emission at 560 nm with a shoulder at 615 nm. In contrast, the solid samples showed emission at significantly longer wavelengths, with a maximum at 630 nm (Figure S2). This is even higher than observed in previous studies, where emission maxima were at 590 to 600 nm [1]. The difference can be attributed to higher dye concentrations leading to the formation of molecular

aggregates, energy transfer processes, and the inner filter effect occurring in optically dense systems. A significant reduction in fluorescence intensity was observed if PU is involved (Figure S2).

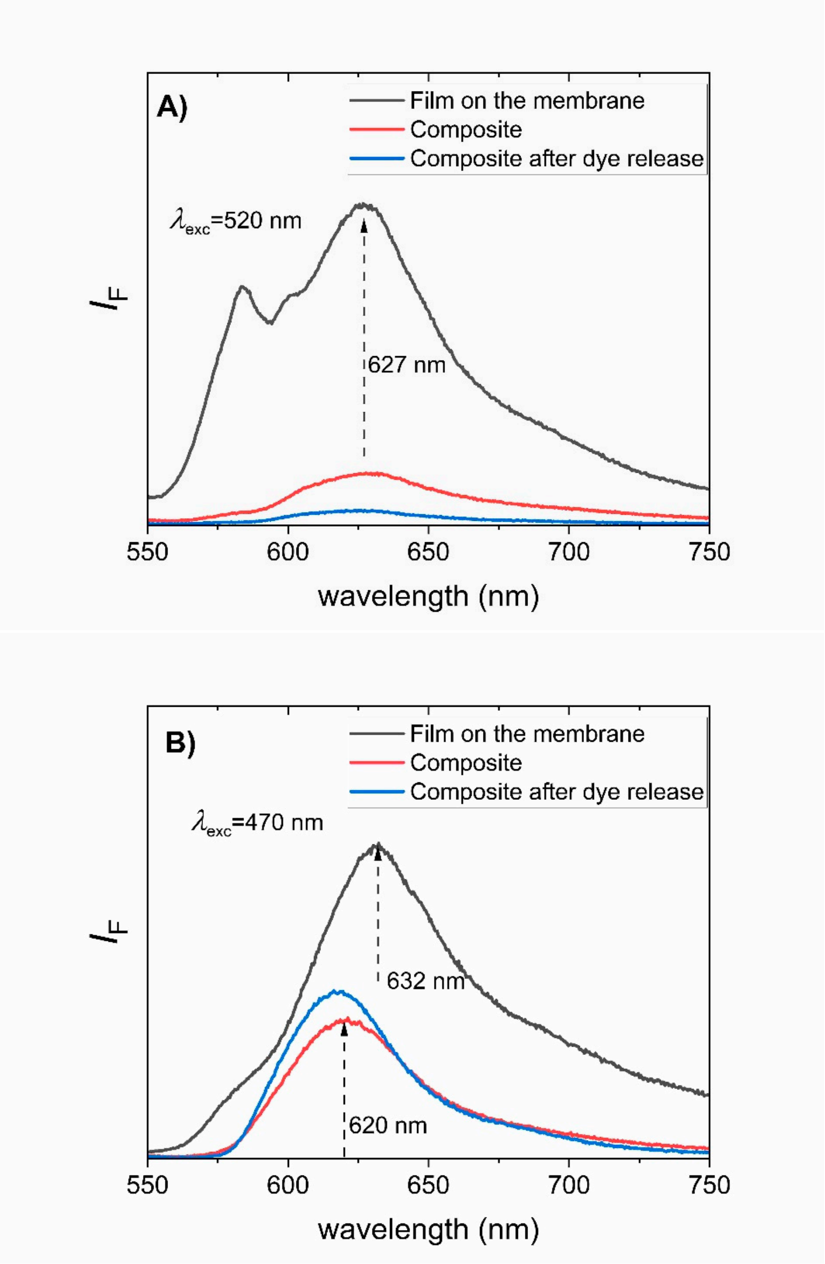

Figure S2. Emission spectra of the OC/PhB film, and its composite with PUC/PhB before and after the release of the dye. The excitation wavelength was 520 nm **(A)** and 470 nm **(B).**

Excitation in the 500–530 nm range is similar to that observed for diluted PhB solutions, suggesting that the high emission from the film can be attributed to a relatively large amount of unaggregated dye molecules (Figure S3 A). The red-shifted fluorescence can be assigned to the inner filter effect among the interactions between photoactive species and molecular aggregates. Excitation at shorter wavelengths, such as 470 nm, can be assigned to molecular aggregates. Such species significantly affect the spectra of the composite before (Figure S3 B) and mainly after the release of the dye (Figure S3 C).

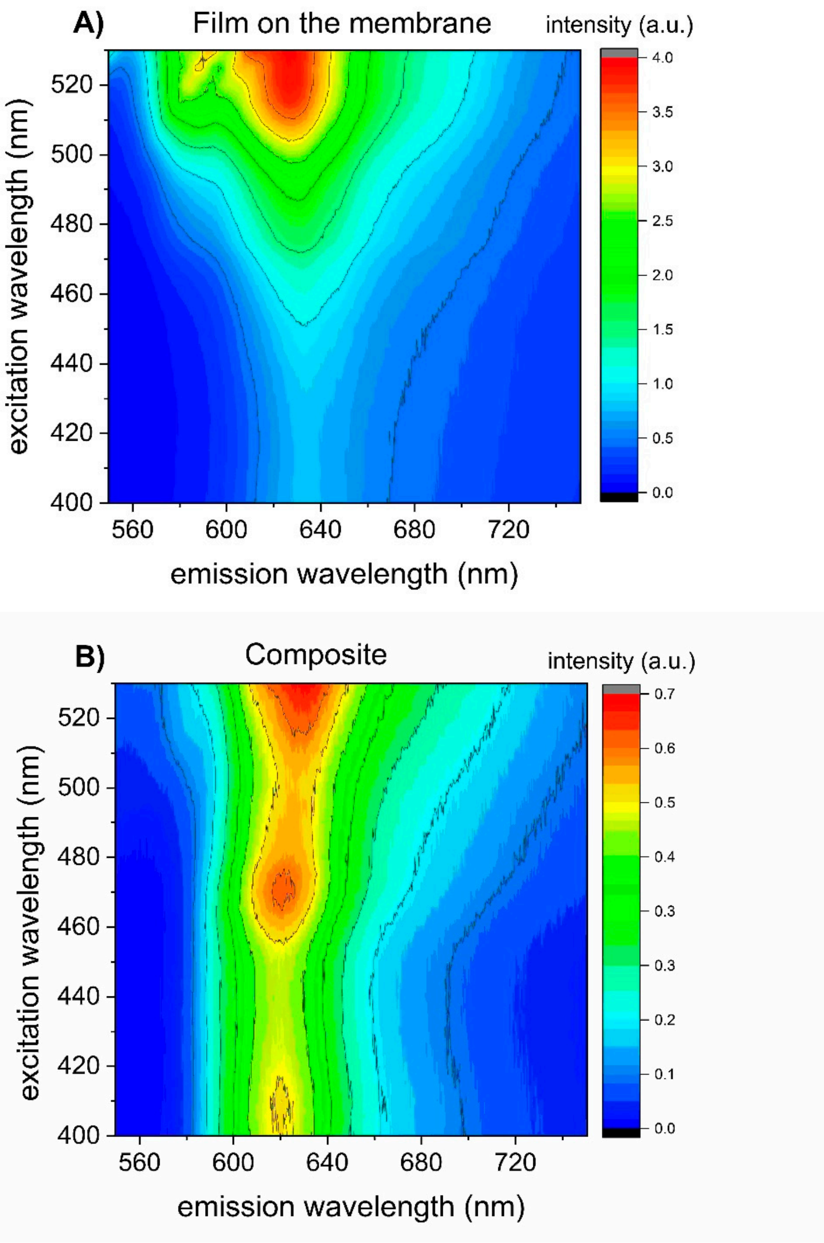
A)
Film on the membrane
intensity (a.u.)
4.0
3.5
3.0
2.5
2.0
1.5
1.0
0.5
0.0
520
500
480
460
440
420
400
excitation wavelength (nm)
560
600
640
680
720
emission wavelength (nm)
B)
Composite
intensity (a.u.)
0.7
0.6
0.5
0.4
0.3
0.3
0.2
0.1
0.0
520
500
480
460
440
420
400
excitation wavelength (nm)
560
600
640
680
720
emission wavelength (nm)

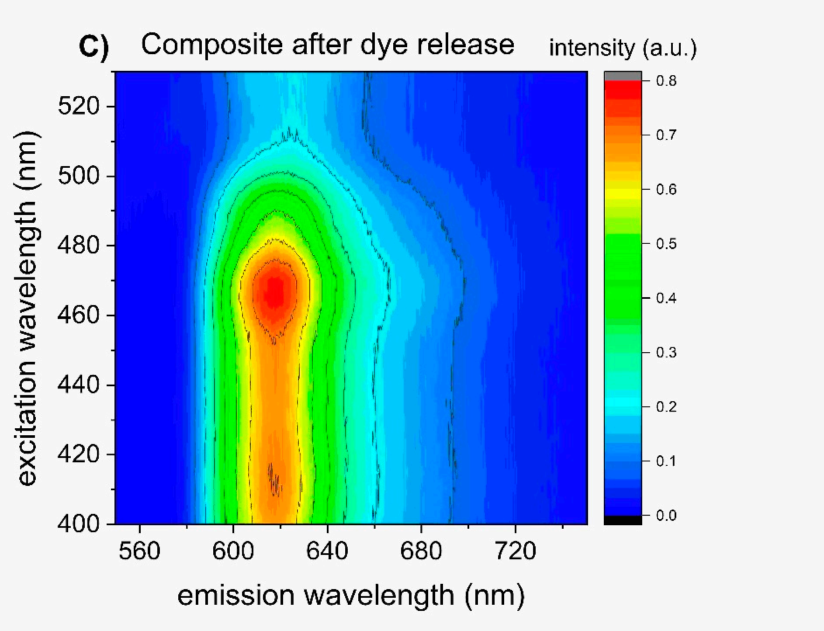

Figure S3. Excitation/Emission 3D plots of the OC/PhB film **(A)**, and its composite with PU before **(B)** and after the release of the dye **(C)**.

Anti-Biofilm Effectiveness of PDI on C. albicans biofilm

The testing different concentrations of PhB (Figure S4) before and after PDI (120 s) on 24-h biofilms of *C. albicans*. Results showed the high effectiveness of 1 and 0.5 mM PhB even in the dark.

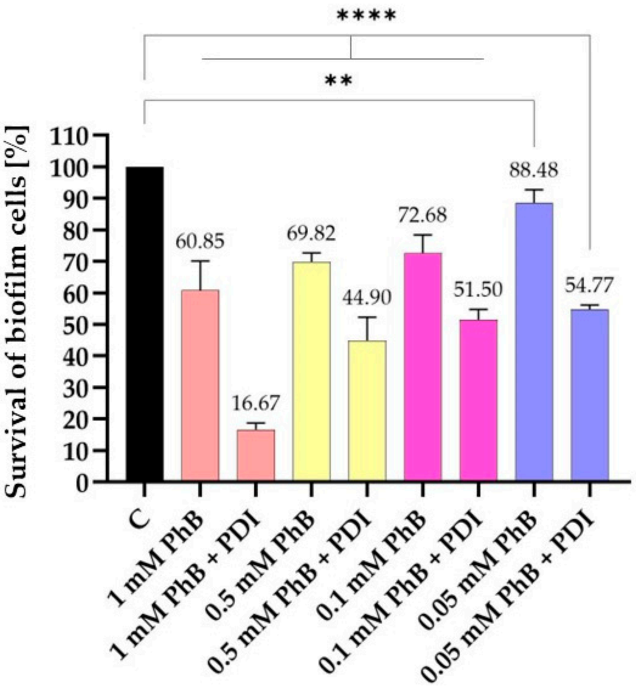

Figure S4. Inhibitory effect of PDI on 24-h biofilm of *C. albicans* SC 5314 after application of different concentrations of PhB with irradiation for 120 s; C-control without PhB. The results were considered to be significant at different *p*-values: $p < 0.01$ (**) and $p < 0.0001$ (****).

Anti-Biofilm Effectiveness of PDI on C. albicans biofilm formed on Nanocomposite

The anti-biofilm activity of nanocomposites was tested on a 24-h biofilm of C. albicans SC5314 on PU discs alone, which represented the control. PUC represented PU modified with hybrid film OC/PhB, with and without irradiation (PDI). All types of PU discs were prepared as described in section 2.2. The preparation of 24-h biofilm of *C. albicans* SC 5314 was the same as in section 2.5. Briefly, an overnight culture of *C. albicans* SC 5314 was harvested by centrifugation (Universal 32 R Hettich Zentrifugen, Tuttlingen, Germany), washing with PBS, and centrifugation. The pellet was resuspended in PBS. The final density of *C. albicans* SC 5314 was adjusted to 4×10^6 cells/mL in MHB supplemented with 2% D-glucose (Centralchem, Bratislava, Slovakia) and 1 mL of suspension was added to 24-well microtiter plate (Sardstedt AG & Co, Germany) with PU discs, then incubated at 37 °C (Thermostatic Cabinet, Lovibond, Germany) for 24 h. The biofilm was then irradiated as described in Section 2.7 for 120 s. The results (Figure S5) show that after irradiation of the composite with PhB, the cell survival was 6.24×10^5 CFU/mL compared to the control growth on PU (4.73×10^6 of CFU/mL). Reduction in the growth of biofilm cells corresponds to almost 90 %.

The normality of the data was determined by the Shapiro-Wilk test and further analysis of the effectiveness of PDI, the Kruskal-Wallis nonparametric test was used. Dunn's comparison test was used to determine significant differences between the groups (Graph Pad, San Diego, CA, USA). Differences were considered significant at various *p*-values: $p < 0.05$ (*), p < 0.01 (**), $p < 0.001$ (***), and $p < 0.0001$ (****).

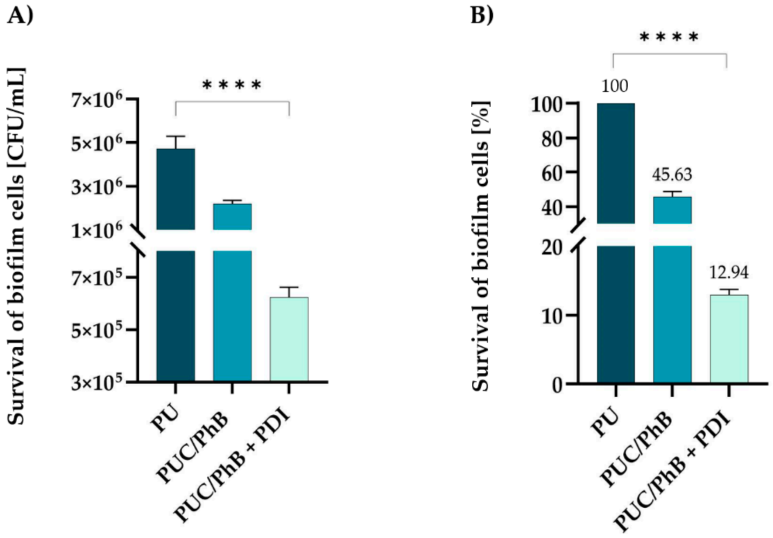

Figure S5. Inhibitory effect of nanocomposite on a 24-h single biofilm of *C. albicans* SC5314 shown as CFU/mL **(A)**; and percentage of surviving cells before and after PDI (120 s) **(B)**. **PU** represents the control samples without irradiation, **PUC/PhB** represents the toxicity control without PDI, and **PUC/PhB + PDI** is the sample after irradiation. The results were considered to be significant at different *p*-values: $p < 0.05$ (*), $p < 0.001$ (***), and $p < 0.0001$ (****).

Reference

1. Dadi, N.C. teja; Dohál, M.; Medvecká, V.; Bujdák, J.; Koči, K.; Zahoranová, A.; Bujdáková, H. Physico-Chemical Characterization and Antimicrobial Properties of Hybrid Film Based on Saponite and Phloxine B. *Molecules* **2021**, *26*, 325, doi:10.3390/molecules26020325.
